# Ontology of doctor and patient relationship and bioethics: from Aristotle’s teleology to Pellegrino’s philosophy of medicine

**DOI:** 10.1007/s11019-024-10239-2

**Published:** 2024-11-27

**Authors:** Nuno Ribeiro Ferreira, Américo Pereira, Rui Nunes

**Affiliations:** 1 Bioethics Centre, Faculty of Medicine, University of Porto, Alameda Prof. Hernâni Monteiro, Porto, 4200-319 Portugal; 2 Faculty of Human Sciences, Catholic University of Portugal (UCP), Palma de Cima Campus, Lisboa, 1649-023 Portugal

**Keywords:** Ontology, Philosophy of medicine, Bioethics, Doctor-patient relationship

## Abstract

Some philosophical and metaethical theories have tried to provide a fundamental background for bioethics but miss the fundamental question about what medicine is, its nature and its end. We argue that the philosophy of medicine, through the development that Edmund Pellegrino and David Thomasma gave to this field of study, allied with Aristotle’s practical and teleological ethics, can provide an ontological background for bioethics beyond the tradition of principles and deontology, with particular emphasis on the uniqueness of the doctor-patient encounter. Some difficulties and criticisms of this ontological model are also examined.

## Introduction

The multiplicity of philosophical and theoretical backgrounds for bioethics, from the Hippocratic deontological code to Beauchamp and Childress’ principlism (2019) and others, all aim to provide the fundamental and ulterior justification for the right and good of medical practice. However, these schools of thought seem to miss a fundamental feature that we argue to be essential to building a solid background for bioethics: the necessity to establish this background within the nature of medicine itself that is expressed in doctor and patient clinical encounter. We specifically call this model an ontology of doctor and patient relationship because we argue that it is the clinical encounter that best expresses the nature of medicine in terms of a practical art of healing and taking care, oriented towards the good of a particular patient. The ontology of doctor and patient relationship may be the foundational background that philosophy of medicine can provide to bioethics, and we follow Pellegrino and Thomasma’s conception of the philosophy of medicine, their emphasis on the phenomenology of the clinical encounter and the telos of medicine, as well as Aristotle’s concepts of ethic, praxis, virtues, and excellences which are the constituting features of his teleological ethics. The ontology of doctor and patient relationship is not disconnected from other previous, and not rarely conflicting ontologies that much contributed to the theoretical discussion within the philosophy of medicine, such as the phenomenology of disease, the ontology of the body and others. Our intention is not to provide a single explanatory model. Instead, the doctor-patient relationship ontology embraces some notions from the phenomenological, humanistic and existential perspectives articulated with Pellegrino and Aristotle theories to provide a praxiological background for medical ethics. Firstly, we will discuss the possibility of such kind of ontology based on the Pellegrino’s philosophy of medicine and Aristotle’s ethics. Secondly, we will try to show if such an ontology based on the intrinsic nature of the clinical encounter can effectively provide strong and solid background for medical ethics.

## The possibility of an ontology of the doctor–patient relationship provided by the philosophy of medicine: from Aristotle to Edmund Pellegrino

The traditional Aristotelian distinction between theoretical virtues (*dianoetikai*) and ethical virtues (*ethikai*) has put in evidence the idea of *ethos*, which means character or habit (Aristotle 2009; pp. 21–22). The fundamental meaning of *ethos* seems to constitute a vector that goes deep into human interiority - the ethical excellencies are excellencies of the human character. Aristotle’s appeal for *ethos* as a topographical nucleus or habitat (also etymologically related to *ethos*) establishes the core of all human possibility. This is the so-called ethical domain, from which ethics is derived etymologically. As a disposition of the character, actualized through excellency, virtues directly support and become intrinsically part of the act itself, thus the only way to be virtuous is to do virtuous things. In medicine, it is by the virtuous disposition of character that emerges in the praxiological doctor-patient relationship that a doctor fulfils his duty of healing and taking care, guided by the end of medicine which is the good of a particular patient. This good is not an abstract and undefined entity. It is an end in itself, something to achieve, something “that at which all things aim” (Aristotle 2009; p. 3). Also, one must not forget the traditional Aristotelian dialectic tension between actuality (*energeia*) and potentiality (*dynamis*) of reality and its repercussion on ethics (Unlu 2022). The dominion of *ethos* appears as the human potency to be that must become an act, in order to be. The motor of this ethical movement is the good that we aim to achieve by acting. *Ethos* is all about the actualization of the potentiality within; any attempt to establish a foundational background for medical ethics must answer this fundamental question: what do we want to do with our human potential? It is through action that we become ethical beings in the realm of praxis. Therefore, praxis is where any philosophy of medicine should radicate the fundamental ontology that supports the ethics of the medical act by fulfilling the potentiality of medicine as an art of healing and taking care, teleologically oriented towards an end. The enormous variability of doctor and patient relationship has repercussions in what we want to achieve through the medical act. The end can be the cure, the mitigation of suffering or simply taking care of those afflicted by disease. But, within the plurality of doctor and patient relationship, the potentiality of the act aligned with the best way to proceed, through the excellency of the act, must be oriented towards what constitutes the good of a particular patient.

Edmund Pellegrino and David Thomasma were pioneers in the field of philosophy of medicine (2018), contributing significantly to its conceptual clarification, object of study, methodological inquiry (1980; 1998) and its relationship with other disciplines such as the humanities, philosophy of science, and bioethics. Furthermore, Pellegrino elucidated the relationship between philosophy of medicine and bioethics, asserting that bioethics inherently requires the philosophy of medicine due to the metaphysical, epistemological and phenomenological nature of the fundamental questions it raises (2001a). To address these questions, one must first examine Pellegrino’s theory of beneficence-in-trust, the nature of the doctor-patient relationship and Aristotle’s ethics of praxis to craft an ontological theory for medical ethics based on doctor and patient relationship.

Pellegrino’s philosophy of medicine can be characterized by its dialectical approach: it is simultaneously pluralistic and unitary, rational yet practically based, and both ontological and value-laden.

Firstly, its pluralism arises from addressing not only the significant changes in medical sciences and technology that alter the practice of medicine but also the deeply personal experience of disease and the varied contexts of clinical encounters. Furthermore, to establish itself as a foundational field, philosophy of medicine must develop a theory that integrates the diverse disciplines it intersects with, such as the philosophy of science, humanities, sociology, and ethics (Engelhardt Jr 1990). This integration serves as both the raison d’être and the foundational basis of the discipline.

Secondly, the rational aspect of Pellegrino’s philosophy is evident in the analytical approach to the clinical encounter between doctor and patient. This approach is grounded in the tangible realities and nuances of medical practice, thereby adopting a praxiological and realistic methodology (Thomasma 1990).

Finally, Pellegrino’s work is rooted in the very nature of medicine as *‘techné iatriké’* — a healing practice. Medicine possesses intrinsic values that are tied to its essence, focusing on the goals of caring for and healing those in need (Pellegrino 2001a). These intrinsic values become manifest in the clinical setting of healing and caring.

The philosophy of medicine, as conceptualized by Edmund Pellegrino and David Thomasma, offers a strong foundational background by asserting that the ethics of medicine originates from the discipline’s very nature. From this nature, principles, virtues, and obligations naturally emerge, intrinsically linked to medicine and its ultimate purposes — healing and caring. Therefore, Pellegrino’s philosophy of medicine is teleological, oriented towards its ends, yet it also embraces realism, essentialism, and pragmatism. This approach is grounded in the phenomenology of the doctor-patient relationship (Pellegrino 2001a). Recognizing the ends of medicine highlights the inherent good in medical actions (Pellegrino 2001b). Pellegrino acknowledges the influence of Aristotle’s teleological and pragmatic virtue theory, noting his theory’s Aristotelian/Thomistic resonance (2001a) particularly in the concept of virtue and *telos* as defined classically by Aristotle. In his *Nicomachean Ethics*, Aristotle distinguishes between ends that are sought for their own sake and those pursued as means to other ends (2009). The ultimate end, desired for its own nature, is crucial in guiding life decisions. Virtue, characterized as a quality of the character, aims towards this end through excellence. Thus, we embody virtue by practicing excellence or actualizing our potential, dedicated to a specific purpose. Pellegrino´s approach to doctor and patient relationship finds in Aristotle´s virtue theory and teleologic ethics, the foundations for bioethics that is intrinsically related to medicine own nature and ends, deeply committed to patient´s own good.

For Pellegrino and Thomasma, medicine’s essence is captured as *techné iatriké*, a practical science governing the art of healing, where the ultimate goal is the patient’s well-being. The ethical foundation of medicine emerges intrinsically from the medical act itself, manifesting within the dynamics of the doctor-patient relationship. Thus, they argue, ethics are not merely external principles imposed upon medical practice (1981) Critiqued for their approach’s perceived excessive individualism and strict adherence to Aristotelian principles, Pellegrino and Thomasma countered by advocating for a balanced, realistic approach rooted in medicine’s inherent nature. This approach is open to an eclectic methodology, incorporating both phenomenological and hermeneutical strategies without fully committing to their respective ontological frameworks (Thomasma and Pellegrino 1987) but building a new one based on medicine´s own nature and essence.

In our view, the methodology of Pellegrino and Thomasma, along with their foundational emphasis on medicine’s core nature and the significance of the doctor-patient interaction, presents a compelling ontology. Their theory does not limit itself to a singular philosophical method but rather embraces a variety of insights from across the philosophical spectrum. It does not exclude important contributions from existentialism and the situational nature of human beings, as beings in the world, it is aware of the social and global determinants of health and healthcare and it is not isolated from the debates on the concepts of health and disease, which certainly have deep repercussions in the type of relationship we establish in the medical scenario. Although we did not develop other compelling ontologies that much influence the doctor and patient relationship, we think Pellegrino´s approach has the original capacity to overtake what is external, instrumental and not directly related with medicine´s nature and formal conditions of medical act, therefore focusing on the ontology of the clinical encounter, from which the inherent ethics of medicine emerge. Pellegrino and Thomasma´s methodology is not a rigid and systematized theory attempting to synthesize all contributions from disparate fields into one. Instead, it stands as an open ontology, primarily concerned with the intrinsic qualities of medicine and the act of medical practice.

## Can the doctor and patient centered ontology provided by the philosophy of medicine serve as the foundational framework for bioethics?

Pellegrino and Thomasma’s philosophy of medicine places paramount importance on the clinical encounter, viewing medicine as a scientific discipline deeply rooted in the human condition (1987). For them, the doctor-patient relationship is the cornerstone of the philosophy of medicine and embodies medicine’s inherent morality. The direct interaction between patients in need and a doctor committed to assisting them forms a distinct and definitive act that underpins the very purpose of medicine’s existence. Consequently, this interaction becomes the *locus ethicus* of medicine, guided by its telos—the patient’s well-being (Pellegrino 2001a). This encounter, while existential, situational, and tangible, serves as the *primum movens* in the doctor-patient relationship, inherently linked to medicine’s essence. We argue that this ontological approach identifies the genesis of values, goods, and ethics within the relationship itself—stemming directly from medicine’s nature—rather than from external, culturally or socially constructed norms, abstract principles or deontological codes. According to Pellegrino, this internal morality does not derive its authority from physicians or ethical codes but from the objective moral order intrinsic to medicine; it integrates insights from pragmatic and virtue theories inspired by Aristotle and remains open to contributions from humanities, history, sociology, and physical sciences. These disciplines enrich our understanding of the moral life’s existential aspects and support the clinical encounter’s realistic and praxiological objectives (Pellegrino 2001a, b). David Thomasma further emphasizes what he terms the “formal conditions of medicine” rooted in its praxis nature. From this foundation, the philosophy of medicine could potentially establish the comprehensive framework for medical ethics, suggesting a profound interconnection between the clinical encounter, the practice of medicine and ethical considerations (Thomasma 1980, 1990). The central role of the physician-patient relationship highlights the tangible reality of illness as an existential fact that causes suffering and vulnerability. Patients entrust their well-being to doctors, who are tasked with addressing the health needs arising from illness (Thomasma 1990). To serve the patient’s good, physicians must act medically, possessing virtues that enable the realization of this goal. Decision-making in medicine, therefore, should be guided not only by principles but also by virtues, which are crucial in achieving medicine’s ultimate purpose.

Both physicians and patients are required to embody virtues that facilitate the pursuit of good within the medical act. Physicians are expected to engage altruistically, prioritizing their patients’ welfare in the healing process and always guided by the scientific knowledge that legitimates medicine; patients, in turn, affirm their autonomy and dignity by placing their trust in the physician’s scientific and humanistic judgment. As noted by Pellegrino and others (Gillon 1985) beneficence may sometimes conflict with autonomy, and the principle of autonomy is not immune to criticism when it precludes trust in doctor-patient relationship (Genuis 2021). Pellegrino’s principle of beneficence-in-trust suggests that autonomy is expressed through trust in the physician’s virtuous actions—honesty, integrity, and a commitment to the patient’s best interests—and that physicians must trust their patients’ dedication to self-care and healing. This mutual trust, rooted in the virtuous character of both doctor and patient, underpins the doctor-patient relationship and is essential for realizing the patient’s well-being (Thomasma 1990). This principle is not a predetermined approach to clinical encounters but emerges from the unique relationship itself, enabling ethical commitment.

Medicine cannot fulfill its purpose without trust, a foundational element from which values, virtues, principles, and rules are derived. The principle of beneficence-in-trust navigates the potential conflict between differing worldviews and value hierarchies, affirming patient autonomy through trust in physicians and establishing trust in patients as essential for achieving the patient’s ultimate good.

Pellegrino and Thomasma anchor their philosophy of medicine in the doctor-patient relationship, epitomizing medicine’s nature as both a practical art and science of healing. This approach aligns with Aristotle’s ethical theory, which emphasizes practical action towards achieving the good as an end goal (Anagnostopoulos 2009). Human action is the basis of ethical agency, necessitating a practical rather than theoretical approach to ethics. Aristotle’s concept of *phronesis*, or practical wisdom, finds a contemporary parallel in the specific dynamics of doctor-patient interactions, where ethical action aims at the good realized in everyday clinical encounters. Such dispositions, embodying excellence in both doctors and patients, fulfill the ultimate purpose of these encounters: the patient’s healing.

Thus, we contend that a foundational framework for medical ethics can be established, grounded in a pragmatic, realistic and teleological ontology of doctor and patient relationship. Firstly, because it is rooted on the nature of medicine as a practical science of healing and an art of caring, we believe it constitutes a primary ontology of medicine. Secondly, because of the teleological aspect of the doctor-patient encounter, oriented towards a specific end, it expresses an axiology of medical act, because there are values, born within the clinical encounter, necessary for the prosecuting of the end, and means of the medical act. Thirdly, because the ontology and axiology are brought within the diversity and pluralism of clinical encounters, in a pragmatical soil that is free from external and operational aspects, it offers, in our view, a potential solution for the problems arising from ethical dilemmas and conflict of principles. For these reasons we argue that the ontology and axiology of medicine that we foresee in Aristotle’s ethics and in Pellegrino’s philosophy of medicine can constitute a foundational framework for medical ethics.

## Some critiques of the philosophy of medicine, the internal morality of medicine and the doctor and patient ontological approach to bioethics

The critique of establishing an ontological foundation for bioethics based on Pellegrino´s philosophy of medicine and Aristotle’s ethics unfolds from three main arguments: one more general questioning the nature and possibility of the independent field of the philosophy of medicine and two more specific challenging foundationalism and ontology in a diverse and postmodern context and scrutinizing the emphasis on the doctor-patient encounter as the cornerstone of medical philosophy and ethics.

Arthur L. Caplan disputes the autonomy of philosophy of medicine, suggesting it is merely a sub-discipline of the philosophy of science. He argues that philosophy of medicine’s focus on epistemological and metaphysical questions diverges fundamentally from bioethics’ normative aspirations (1992). This critique extends to deny not only the field’s independence but also its capability to underpin bioethics, citing intrinsic differences between the disciplines.

Conversely, Wulff defends the viability of philosophy of medicine as a distinct area, emphasizing its grounding in medical practice and its integration of philosophical insights. From this perspective, philosophy of medicine is seen as a significant medical specialty that enriches rather than detracts from the broader philosophical discourse (Wulff 1992).

These discussions reveal the contentious nature of philosophy of medicine’s status and its contributions to bioethics, underscoring ongoing debates about the field’s epistemological boundaries, its foundational premises, and the practical implications of adopting an ontological approach grounded in the clinical experience.

As Thomasma highlights, anti-foundationalist critiques often stem from philosophical traditions rooted in objectivism, moral relativism, deconstructionism, and neopositivism, drawing significantly from the works of Foucault, Deleuze and Derrida (1997). Foucault’s analyses have profoundly affected how we understand the interplay between clinical medicine and the frameworks of power, politics and society, reshaping clinical epistemology over time (Suijker 2023). Similarly, MacIntyre emphasizes the role of pluralism and social constructivism within the moral sphere (2007), while Putnam critiques the objectivity of morality and ontological realism (2005), questioning the feasibility of establishing a definitive ethical foundation.

These discussions challenge the possibility of an ultimate ontological grounding for ethics, suggesting that ethical inquiry is driven more by the need for clarification and practical problem-solving (Nath 2019), rather than by the pursuit of absolute truths or universal principles, which are seen as products of social and collective constructs.

Importantly, ontology is not anchored in abstract notions of health and disease but is deeply embedded in the tangible realities of the clinical environment. This approach recognizes that ethical principles are inseparable from the lived world, emerging directly from the practice of medicine and the experiences of patients and doctors. The lived experience is the basis for bioethical comprehensibility and meaning. Influenced by Pellegrino, Thomasma and Aristotle, this perspective acknowledges the complexities of moral diversity, multiculturalism, and the social determinants of health, integrating these factors into the fabric of medical practice (Reynolds 2018; Kopelman 2019). As we have previously discussed, Pellegrino´s defence of an internal morality of medicine, emerging from medicine´s nature and from the doctor and patient relationship imposes some problems concerning the pluralism and diversity of the clinical encounters, the multiplicity of what constitutes the good for the patient and the constant evolution of the ends of medicine, due to external factors that Pellegrino tends to undervalue, according to some critics(Veatch 2001). In our view, Pellegrino’s attempt to build a morality of medicine directly driven from the essence of the medical encounter is guided by the urge to differentiate what is constant and foundational in medicine from what is external, social and cultural determined and therefore, always evolving. Pellegrino´s philosophy acknowledges what is external to medicine and the influences in everyday practice but do not establish foundations for bioethics in these external factors, but rather in what is definitional and essential.

The debate between realistic and antirealist views of health and disease, and their implications for the philosophy of medicine and bioethics (Simon 2008; Khushf 1997) illustrates that the ontological exploration is not an isolated endeavor to lay down ethical foundations. Instead, it arises from the diverse and rich discourse within the philosophy of medicine, aiming to establish a grounded, comprehensive framework for medical ethics that fully embraces the multifaceted nature of medical practice. The debate about the nature of health and disease does not preclude the possibility of an ontological model based on doctor and patient relationship. The ontological model embraces the question raised by the debate into a broader conceptualization about the nature of the clinical encounter and the importance of definitions and its repercussions in the medical act.

In discussing the ontological approach to bioethics within the philosophy of medicine, we align with Pellegrino’s perspective that this philosophy fundamentally seeks to understand the phenomena and nature of everyday medical practices, aiming to solidify bioethics on robust, eclectic, and methodologically sound grounds. The post-war evolution of philosophy towards hermeneutical, existential and phenomenological reflections has emphasized the significance of narrative medicine and the vivid experiences of suffering, identity and finitude that doctors and patients encounter daily (Pellegrino 1998). While insights from laboratory sciences, epidemiology, public health, and socioeconomics are invaluable, it is the clinical encounter between doctor and patient that truly defines medicine as a practical science and art, aiming towards specific goals and ends. These multiple and compelling ontologies offers different views about medicine and bioethics and do not preclude the ontological model based on the doctor and patient clinical encounter.

The pluralistic and diverse nature of modern societies reveals challenges posed by universal moral theories that fail to acknowledge the nuances of specific cultures or societies, questioning the very possibility of a common morality and the establishment of universal principles and moral obligations. Critics like Wildes have pointed out the need for the philosophy of medicine to consider the social determinants of health and disease, rather than solely basing itself on the intrinsic nature of medicine as revealed through the clinical encounter (2001). Furthermore, Pellegrino’s emphasis on the doctor-patient relationship has been critiqued for potentially narrowing the scope of discussion to exclude the diversity of such relationships in a multicultural and heterogeneous world, as well as the specific institutional contexts shaped by resource allocation (Stempsey 2004). Others have highlighted the potentially overlooked role of medicine in a global context, emphasizing health promotion and self-care that extend beyond the doctor-patient dynamic (Whitbeck 1981). Additionally, the influence and responsibility of physicians within the social structures where they practice, which can impact medical practices negatively, have been underscored (Ozar 1985).

It’s essential to remember that the clinical encounter itself constitutes a fundamental social interaction, potentially the foundation for broader medical relationships. Pellegrino and Thomasma view the doctor-patient relationship as the cornerstone of clinical medicine, where the encounter’s nature, revealing patient vulnerability and physician solicitude, brings to light the essence of medicine’s goals, purposes, and aims. While the social and institutional determinants of medicine are acknowledged, such as the cooperative nature of modern medicine and the support of institutions, these factors do not form the pillar from which the ontology and axiology of medicine is derived. Instead, their value is seen as external to the core nature of medicine, being operational and instrumental (DeCamp 2019).

Following Aristotle’s transition from ethical to the political realm, Pellegrino directs us from individual virtues to societal values, suggesting a pathway from personal ethics within the clinical encounter to broader societal ethical considerations (2001b).

Thus, while acknowledging critiques, the ontological model seeks to ground medical ethics in the praxis of medicine as a *techné iatriké*, focusing on the clinical setting as the essential nucleus of medicine and its intrinsic aims and purposes. This perspective advocates for a medical ethics framework that is deeply rooted in the nature and practice of medicine, providing a comprehensive referential basis for bioethics.

The ontological model does not endeavor to create static *à priori* principles. Rather, the aim is to establish a robust foundation for medical ethics, deeply rooted in the essence of medicine’s practice. By anchoring ethics in the ontological heart of medicine, within its clinical praxis and inherent goals, a fertile soil is established from which a comprehensive theory of medical ethics can grow. It is through an exploration of medicine’s nature as praxis that we seek to identify the ultimate reference for bioethics. This approach ensures that ethical frameworks are intimately connected to the practical and purposeful actions of medicine, offering a dynamic and contextually relevant guide for ethical decision-making in the medical field.

## Conclusion

The philosophy of medicine offers a critical examination of the theory and practice of medicine, its essence, and ultimate objectives. It promises to provide a foundational backdrop for enriching the practice of medicine with inherent good. The prevailing philosophical and metaethical theories often miss crucial ontological aspects, tending towards operational effectiveness, resolution of dilemmas, and moral relativism instead. The ontological perspective, significantly inspired by Pellegrino and Thomasma’s views on the philosophy of medicine, along with Aristotle’s practical and teleological ethics, underscores the significance of the clinical encounter. This encounter defines the essence of the medical act, aligning ethics closely with medicine’s ultimate goals as manifested within these interactions.

The pursuit of good in medical practice is intricately linked to the doctor-patient relationship, portraying medicine as a practical art dedicated to healing. For bioethics to be truly meaningful, it must connect intimately with the nature of medicine and its primary objective: the well-being and healing of the patient. While factors such as institutional frameworks, health systems, interdisciplinary healthcare relationships, and the socio-cultural milieu undoubtedly influence medical practice, their significance, though substantial, remains instrumental and externally imposed. The distinct and intimate doctor-patient relationship activates the values, virtues, and methods necessary to fulfil the telos of medicine, providing a critical ethical foundation for all medical actions.

## References

[CR1] Anagnostopoulos, Georgios. 2009. *A Companion to Aristotle*. Edited by Georgios Anagnostopoulos. Wiley. 10.1002/9781444305661

[CR2] Aristotle. 2009. *The Nicomachean Ethics Trans. David Ross*. Edited by Lesley Brown. Oxford University Press.

[CR3] Beauchamp, Tom, James Childress. 2019. *Principles of Biomedical Ethics*. 8th ed. New York: Oxford University Press.

[CR4] Caplan, Arthur. 1992. Does the philosophy of Medicine Exist? *Theoretical Medicine* 13: 67–77. 1604434 10.1007/BF00489220

[CR5] DeCamp, Matthew. 2019. Toward a pellegrino-inspired theory of value in Health Care. *Theoretical Medicine and Bioethics* 40(3): 231–241. 10.1007/s11017-019-09486-9 31289967 10.1007/s11017-019-09486-9

[CR6] Engelhardt Jr, H.Tristam. 1990. The birth of the Medical Humanities and the rebirth of the philosophy of Medicine: The vision of Edmund D. Pellegrino. *The Journal of Medicine and Philosophy* 15: 237–241. 2203863

[CR7] Genuis, Quentin, I. T. 2021. A genealogy of autonomy: Freedom, Paternalism, and the future of the doctor-patient relationship. *Journal of Medicine and Philosophy (United Kingdom)* 46(3): 330–349. 10.1093/jmp/jhab004 10.1093/jmp/jhab004 PMC8188809 33948633

[CR8] Gillon, Raanan. 1985. Beneficience: Doing good for others. *British Medical Journal* 291: 44–45. 3926060 10.1136/bmj.291.6487.44 PMC1416194

[CR9] Khushf, George. 1997. Why Bioethics need the philosophy of Medicine: Some implications of reflection on concepts of Health and Disease. *Theoretical Medicine* 18: 145–163. 9129398

[CR10] Kopelman, Loretta M. 2019. On Pellegrino and Thomasma’s admission of a Dilemma and Inconsistency. *The Journal of Medicine and Philosophy* 44(6): 677–697. 10.1093/jmp/jhz027 31609420 10.1093/jmp/jhz027

[CR11] MacIntyre, Alasdair. 2007. *After Virtue: A Study in Moral Theory*. Third edition. London: Duckworth.

[CR12] Nath, Rajakishore. 2019. Can Ethics be without Ontology? Wittgenstein and Putnam. *Philosophia (United States)* 47(4): 1215–1225. 10.1007/s11406-018-0035-1

[CR13] Ozar, David. 1985. Social Ethics, the philosophy of Medicine and Professional Responsabiliity. *Theoretical Medicine* 6: 281–294. 4060090 10.1007/BF00489730

[CR14] Pellegrino, Edmund. 1998. What the Philosophy of Medicine is. *Theoretical Medicine and Bioethics* 19: 315–336. 10.1023/A:1009926629039 9865137 10.1023/a:1009926629039

[CR15] Pellegrino, Edmund. 2001a. Philosophy of Medicine: Should it be teleologically or socially construed? *Kennedy Institute of Ethics Journal* 11(2): 169–180. 10.1353/ken.2001.0015 11708333 10.1353/ken.2001.0015

[CR16] Pellegrino, Edmund. 2001b. The Internal Morality of Clinical Medicine: A paradigm for the Ethics of the Helping and Healing professions. *Journal of Medicine and Philosophy* 26(6): 559–579. 11735050 10.1076/jmep.26.6.559.2998

[CR17] Pellegrino, Edmund, and David Thomasma. 1981. *A philosophical basis of Medical Practice*. Oxford University Press.

[CR18] Putnam, Hilary. 2005. *Ethics without Ontology*. Cambridge: Harvard University Press.

[CR19] Reynolds, Joel Michael. 2018. Renewing Medicine’s Basic concepts: On ambiguity. *Philosophy Ethics and Humanities in Medicine* 13(1). 10.1186/s13010-018-0061-4 10.1186/s13010-018-0061-4 PMC6032601 29973289

[CR20] Simon, Jeremy. 2008. Constructive realism and medicine: An Approach to Medical Ontology. *Perspectives in Biology and Medicine* 51(3): 353–366. 18723940 10.1353/pbm.0.0032

[CR21] Stempsey, William. 2004. The philosophy of Medicine: Development of a Discipline. *Health Care and Philosophy* 7: 243–251. 10.1007/s11019-004-6469-1 15679016

[CR22] Suijker, Chris A. 2023. Foucault and Medicine: Challenging normative claims. *Medicine Health Care and Philosophy* 26(4): 539–548. 10.1007/s11019-023-10170-y 37747687 10.1007/s11019-023-10170-y PMC10725842

[CR23] Thomasma, David. 1980. A philosophy of a clinically based Medical Ethics. Journal of Medical Ethics 6: 190–196. 7463428 10.1136/jme.6.4.190 PMC1154843

[CR26] Thomasma, David, Edmund Pellegrino. 1981. Philosophy of Medicine as the source for Medical Ethics. *Metamedicine* 2: 5–11. 10.1007/BF00886339 11650508 10.1007/BF00886339

[CR27] Thomasma, David, Edmund Pellegrino. 1987. Challenges for a philosophy of Medicine of the future: A response to fellow philosophers in the Netherlands. *Theoretical Medicine* 8: 187–204. 3660282 10.1007/BF00539755

[CR24] Thomasma, David. 1990. Establishing the Moral basis of Medicine: Edmund D. Pellegrino´s Philosophy of Medicine. *The Journal of Medicine and Philosophy* 15: 245–267. http://jmp.oxfordjournals.org/2203865 10.1093/jmp/15.3.245

[CR25] Thomasma, David. 1997. Antifoundationalism and the possibility of a Moral Philosophy of Medicine. *Theoretical Medicine* 18: 127–143. 9129397

[CR28] Unlu, Hikmet. 2022. Dynamis and Energeia in Aristotle’s metaphysics. *European Journal of Philosophy* 30(1): 17–31. 10.1111/ejop.12635

[CR29] Veatch, Robert M. 2001. The impossibility of a Morality Internal to Medicine. *The Journal of Medicine and Philosophy* 26(6): 621–642. 10.1076/jmep.26.6.621.2996 11735053 10.1076/jmep.26.6.621.2996

[CR30] Whitbeck, Caroline. 1981. On the aims of Medicine: Comments on the philosophy of Medicine as the source for Medical Ethics. *Metamedicine* 2: 35–41. 11650506 10.1007/BF00886344

[CR31] Wildes, Kevin. 2001. The Crisis of Medicine: Philosophy and the Social Construction of Medicine. *Kennedy Institute of Ethics Journal* 11(1): 71–86. 10.1353/ken.2001.0008 12166446 10.1353/ken.2001.0008

[CR32] Wulff, Henrik. 1992. Philosophy of medicine- from Medical Perspective. *Theoretical Medicine* 13: 79–85. 1604435 10.1007/BF00489221

